# Predicting acute radiation dermatitis in breast cancer: a prospective cohort study

**DOI:** 10.1186/s12885-023-10821-6

**Published:** 2023-06-12

**Authors:** Yuxiu Xie, Ting Hu, Renwang Chen, Haiyan Chang, Qiong Wang, Jing Cheng

**Affiliations:** Cancer Center, Union Hospital, Tongji Medical College, Huazhong University of Science and Technology, Wuhan, 430022 China

**Keywords:** Radiation dermatitis, Breast cancer, Ferritin, High-sensitivity C-reactive protein, Predictive factor

## Abstract

**Background:**

Acute radiation dermatitis (ARD) is one of the most common acute adverse reactions in breast cancer patients during and immediately after radiotherapy. As ARD affects patient quality of life, it is important to conduct individualized risk assessments of patients in order to identify those patients most at risk of developing severe ARD.

**Methods:**

The data of breast cancer patients who received radiotherapy were prospectively collected and analyzed. Serum ferritin, high-sensitivity C-reactive protein (hs-CRP) levels, and percentages of lymphocyte subsets were measured before radiotherapy. ARD was graded (0–6 grade), according to the Oncology Nursing Society Skin Toxicity Scale. Univariate and multivariate logistic regression analyses were used and the odds ratio (OR) and 95% confidence interval (CI) of each factor were calculated.

**Results:**

This study included 455 breast cancer patients. After radiotherapy, 59.6% and 17.8% of patients developed at least 3 (3+) grade and at least 4 (4+) grade ARD, respectively. Multivariate logistic regression analysis found that body mass index (OR: 1.11, 95% CI: 1.01–1.22), diabetes (OR: 2.70, 95% CI: 1.11–6.60), smoking (OR: 3.04, 95% CI: 1.15–8.02), higher ferritin (OR: 3.31, 95% CI: 1.78–6.17), higher hs-CRP (OR: 1.96, 95% CI: 1.02–3.77), and higher CD3 + T cells (OR: 2.99, 95% CI: 1.10–3.58) were independent risk factors for 4 + grade ARD. Based on these findings, a nomogram model of 4 + grade ARD was further established. The nomogram AUC was 0.80 (95% CI: 0.75–0.86), making it more discriminative than any single factor.

**Conclusion:**

BMI, diabetes, smoking history, higher ferritin, higher hs-CRP, and higher CD3 + T cells prior to radiotherapy for breast cancer are all independent risk factors for 4 + grade ARD. The results can provide evidence for clinicians to screen out high-risk patients, take precautions and carefully follow up on these patients before and during radiotherapy.

**Supplementary Information:**

The online version contains supplementary material available at 10.1186/s12885-023-10821-6.

## Introduction

Breast cancer is the most frequently diagnosed cancer in females in American and China [1, 2]. Significant advances in diagnostic and therapeutic stratification of breast cancer have significantly prolonged survival. As radiotherapy (RT) reduces breast cancer recurrence and death, over 70% of breast cancer patients undergo adjuvant RT following a lumpectomy or radical mastectomy [3, 4]. When radiation kills tumor cells, it also causes an acute reaction in normal skin in the irradiated area. When a patient has severe acute radiation dermatitis (ARD), it not only affects the patient’s quality of life by causing pain, anxiety, and insomnia, but it also causes the RT plan to be delayed or terminated early, reducing the treatment effect [5]. Severe skin damage after RT also destroys the integrity of the skin’s physical barrier and affects the body’s immune function, increasing the risk of local infection [6]. The majority of patients experience moderate-to-severe skin reactions, and up to 8% of patients experience severe moist desquamation with conventional fractionated RT (CFRT) [7, 8].

ARD includes breast erythema and desquamation, exists in progressive states of severity, and occurs within 90 days of treatment. It is reported that ARD is associated with significant late toxicities, such as telangiectasia [9]. At present, multiple guidelines recommend the use of topical corticosteroids, antiperspirant, washing with water and soap to reduce ARD. There are inconsistencies between the recommendations of the current guidelines and further research is needed to establish the best treatment for the prevention and management of ARD [10]. According to several studies, hypo-fractionated radiotherapy (HFRT) and CFRT have the same efficacy in overall survival, disease-free survival, local recurrence and distant metastasis after radical mastectomy for early breast cancer [11, 12]. However, compared with CFRT, HFRT showed a lower incidence of acute pain, breast edema, ARD, fatigue and telangiectasia [13]. Therefore, HFRT will be a better choice for high-risk patients with severe ARD.

ARD, also known as “complex wound,“ is caused by the loss of functional stem cells, changes in endothelial cells, inflammation, and apoptosis and necrosis of epidermal cells [14]. Inflammation may play a critical role in normal tissue toxicities. RT contributes to inflammatory cell recruitment as well as to direct tissue injury and also activates proinflammatory cytokines and growth factors, which, in turn, promotes inflammation and cytokine overproduction [15]. One study has shown that elevated high-sensitivity C-reactive protein (hs-CRP) has been linked to a significantly higher risk of skin toxicity [16]. Ferritin is not only an iron storage protein but also a well-known inflammatory marker [17]. Ferritin synthesis is regulated not only by proinflammatory cytokines such as interleukin-1β, tumor necrosis factor-α, and interleukin-6, but it can also act as an enhancer of inflammatory response via nuclear factor-κB activation [18]. In addition, cell damage caused by radiotherapy or inflammation further leads to the leakage of intracellular ferritin, leading to elevated serum ferritin [19]. Serum ferritin levels are also elevated in patients with lung, and breast cancer, indicating a poor prognosis [20, 21].

Lymphocyte subsets have a wide range of biological functions and are frequently used as indicators of the quality of the body’s cellular immune function. There are certain abnormalities in the number and function of immune cells in tumor patients, which manifest as an imbalance in the number of T lymphocyte subsets and functional damage. According to recent research, the radiation-induced lymphocyte apoptosis assay (RILA) has been shown to predict acute breast pain, and CD4 + RILA can predict late radiotherapy toxicity, such as subcutaneous fibrosis and telangiectasia [22, 23]. However, few studies have been conducted to investigate the link between abnormal immune function and ARD severity. This study intends to detect the levels of peripheral blood lymphocyte subsets (CD3 + T cells, CD3+/CD4 + T cells, CD3+/CD8 + T cells, CD4+/CD8 + ratio, natural killer (NK) cells, and B cells) in breast cancer patients before RT to understand the immune function of patients and investigate the impact of immune status on the severity of ARD.

We have demonstrated in a comprehensive review and meta-analysis that BMI, large breast volume, smoking habit, diabetes, and sequential boost and bolus use might predict the risk of severe ARD, and HFRT reduced the risk of severe ARD compared to CFRT [24]. It has been reported that ARD is related to race [16], but the majority of these studies come from North America or Europe with little data from China. Therefore, we designed a prospective cohort study to explore which risk factors can predict the occurrence of ARD in Chinese breast cancer patients.

## Methods

### Study Design and patients characteristics

During the period from August 15, 2020 to January 31, 2022, breast cancer patients with adjuvant RT were consecutively recruited from five treatment teams in the Cancer Center, Union Hospital, Tongji Medical College, Huazhong University of Science and Technology for this prospective single center cohort study. Informed consent was obtained from all individual participants included in the study. The inclusion criteria were predefined: (1) female patients aged 18 and up; (2) new diagnosis of stage I-III breast cancer according to the eighth American Joint Committee on Cancer clinical and pathological staging; (3) breast-conserving surgery (BCS) or total mastectomy; and (4) plan to receive adjuvant radiotherapy to the whole breast or chest wall ± nodal irradiation. The exclusion criteria were as follows: (1) bilateral breast cancer; (2) a supraclavicular node or distant metastasis; (3) previous irradiation; (4) previous or concurrent cancers; (5) concurrent chemotherapy; (6) immediate breast reconstruction after mastectomy; and (7) skin disease or active knot-hoof tissue disease.

The following demographic information was collected from the included patients: age, body mass index (BMI), hypertension, diabetes, smoking status (regular smokers or current smokers), pathological diagnosis, tumor sites, TNM stage, the expression levels of estrogen receptor, progesterone receptor, human epidermal growth factor receptor 2, and Ki67, adjuvant or neoadjuvant chemotherapy regimen, RT plans, surgery type, and targeted therapies (trastuzumab, pertuzumab). BMI was stratified into groups: (1) underweight to normal weight (BMI < 25); (2) overweight (BMI ≥ 25 and < 30); and (3) obese (BMI ≥ 30). Patients who did not receive neoadjuvant chemotherapy were assigned a pathological stage. For patients who received neoadjuvant chemotherapy, the higher clinical or pathological stage was used to reflect the actual status.

### RT and skin toxicity Assessment

RT was usually carried out after 4–6 weeks after the completion of surgery or last chemotherapy. All enrolled patients received intensity-modulated radiation therapy (IMRT). The volume of the target and organs at risk was contoured and defined in accordance with the Radiation Therapy Oncology Group guidelines. RT to the chest wall or whole breast is usually delivered at 50 Gy in 25 fractions of 2 Gy over 5 weeks (CFRT). Patients received supplemental electron beam irradiation on the tumor bed after completion of whole-breast RT.

ARD severity was assessed based on the Oncology Nursing Society (ONS) skin toxicity scale every week during RT and the second week after RT by her attending physician and the followers of this study. If their evaluation results were different, the final evaluation results will be further evaluated by the principal investigator. Because the ONS skin toxicity scale describes ARD symptoms more precisely, including the presence and extent of dry and moist desquamation, it is more useful for toxicity classification than the National Cancer Institute Common Terminology Criteria for Adverse Events (CTCAE) [25]. The ONS skin toxicity scale categorizes skin reactions into six categories: (1) faint or dull erythema, follicular reaction, and/or itching (CTCAE grade 1); (2) bright erythema and/or tender to touch (CTCAE grade 2); (3) dry desquamation with or without erythema (CTCAE grade 1 or 2); (4) a small or moderate amount of wet desquamation (CTCAE grade 2); (5) confluent moist desquamation (CTCAE grade 3); and (6) ulceration, hemorrhage, and/or necrosis (CTCAE grade 4).

### Assay for ferritin, hs-CRP, and lymphocyte subsets

Professional nurses drew blood samples from the veins of all included subjects in the morning before the start of RT. The blood collection process strictly followed standard aseptic procedures. The blood samples were collected in a sterile vacuum test tube and centrifuged at 3000 r/min for 10 min to separate serum. The ferritin assay kit was then used to quantitatively assess serum ferritin levels using chemiluminescent microparticle immunoassay technology on an Abbott Architect 16,200 automated analyzer (Abbott, Abbott Park, IL). Hs-CRP levels were quantified by immunoturbidimetry on a Beckman automatic chemiluminescence analyzer. Flow cytometry was performed to examine lymphocyte subsets using a FACSCalibur flow cytometer (BD Bioscience).

### Statistical analysis

Categorical variables were expressed as percentages, and the chi-square test was used for the theoretical frequency ≥ 5; otherwise, the Fisher exact probability method was used. Histogram, P-P diagram and Q-Q diagram were used to test whether the continuous data were normally distributed. Continuous variables were compared by independent t-test if normally distributed. If continuous variables were non-normal distribution, the nonparametric test was used. Participants were divided into four groups based on serum ferritin quartiles, hs-CRP concentrations, and lymphocyte subset percentages, and a linear trend was used to assess the relationship between those variables and ARD severity. The best cut-off point for continuous predictors was determined by the receiver operating characteristic (ROC) curve. To assess potential ARD risk factors, a univariate logistic regression analysis was used. The variables with significant differences were then included in a multivariate logistic regression analysis to identify independent risk factors for ARD and calculate the odds ratio (OR) and 95% confidence interval for each factor (CI).

A nomogram was then constructed using the factors that were significant in the multivariate logistic regression model. The area under the ROC curve (AUC) was conducted to estimate the discriminating ability of the nomogram and each predictor separately. The calibration curve was used to compare the predicted probability with the observed probability of grade 4 + ARD for consistency evaluation. Finally, in order to illustrate the clinical utility of the nomogram, we drew a decision curve analysis (DCA) diagram to quantify the net benefits under different threshold probabilities.

Using a two-sided test, P < 0.05 indicates that the test results are statistically significant. SPSS version 22.0 and R software were used for all statistical analyses (version x64 4.1.2).

## Results

### ARD severity based on the demographic and clinical characteristics of the patient

The baseline characteristics of the 455 patients are summarized in Table 1. In total, 271 (59.6%) patients and 81 (17.8%) patients were diagnosed with ONS at least grade 3 (3+) and at least grade 4 (4+) ARD, respectively. One patient developed grade 6 ARD during RT, so the RT was interrupted and then transferred to plastic surgery for further treatment. Six patients (1.3%) developed grade 5 ARD at the end of RT or within 2 weeks after RT. Higher proportions of grade 3 + or 4 + ARD were observed in the following subgroups: age ≧ 60 years old, BMI ≧ 25 Kg/m², with hypertension, diabetes, and smoking history. No significant grade 3 + or 4 + ARD differences were observed for pathological type, histopathological grade, tumor stage, ER, PR, HER2, triple-negative breast cancer (TNBC), neoadjuvant chemotherapy, surgery, adjuvant chemotherapy, and anti HER2 targeted therapy.

**Table 1:** ARD by Patient Clinical Characteristics

Variable and Category	No. of patients	≧ 3 grade	*P*	≧ 4 grade	*P*
Total No. of patients	455	271(59.6%)		81(17.8%)	
Age (years)					
< 60	380(83.5%)	218(57.4%)	0.039	62(16.3%)	0.07
≧ 60	75(16.5%)	53(70.7%)		19(25.3%)	
BMI					
BMI:<25	341(74.9%)	189(55.4%)	0.02	42(12.3%)	< 0.001
BMI:25 ~ 30	96(21.1%)	66(68.8%)		28(29.2%)	
BMI: ≧30	18(4%)	16(88.9%)		11(61.1%)	
Hypertension					
No	386(84.8%)	216(56%)	< 0.001	54(14%)	< 0.001
Yes	69(15.2%)	55(79.7%)		27(39.1%)	
Diabetes					
No	405(89%)	228(56.3%)	< 0.001	58(14.3%)	< 0.001
Yes	50(11%)	43(86%)		23(46%)	
Smoking history					
No	428(94.1%)	248(57.9%)	0.004	68(15.9%)	< 0.001
Yes	27(5.9%)	23(85.2%)		13(48.1%)	
Pathological type					
Invasive ductal carcinoma	395(86.8%)	240(60.8%)	0.409	69(17.5%)	0.706
Ductal carcinoma in situ	25(5.5%)	13(52%)		4(16%)	
Others	35(7.7%)	18(51.4%)		8(22.9%)	
Histopathological grade					
NA	28(6.2%)	16(57.1%)	0.254	5(17.9%)	0.768
1	14(3.1%)	5(35.7%)		1(7.1%)	
2	193(42.4%)	113(58.5%)		35(18.1%)	
3	220(48.4%)	137(62.3%)		40(18.2%)	
Tumor stage					
I	127(27.9%)	68(53.5%)	0.197	25(19.7%)	0.47
II	190(41.8%)	121(63.7%)		36(18.9%)	
III	138(30.3%)	82(59.4%)		20(14.5%)	
ER					
Negative	137(30.1%)	75(54.7%)	0.177	26(19%)	0.689
Positive	318(69.9%)	196(61.6%)		55(17.3%)	
PR					
Negative	175(38.5%)	99(56.6%)	0.327	35(20%)	0.378
Positive	280(61.5%)	172(61.4%)		46(16.4%)	
HER2					
Negative	336(73.8%)	199(59.2%)	0.829	57(17%)	0.486
Positive	119(26.2%)	72(60.5%)		24(20.2%)	
TNBC					
Negative	372(81.8%)	228(61.3%)	0.137	67(18%)	0.875
Positive	83(18.2%)	43(51.8%)		14(16.9%)	
Ki67					
Ki < 14%	98(21.5%)	60(61.2%)	0.729	18(18.4%)	0.882
Ki > = 14%	357(78.5%)	211(59.1%)		63(17.6%)	
Neoadjuvant chemotherapy					
No	313(68.8%)	187(59.7%)	0.918	56(17.9%)	0.999
Yes	142(31.2%)	84(59.2%)		25(17.6%)	
Surgery					
BSC	131(28.8%)	81(61.8%)	0.598	23(17.6%)	0.999
Mastectomy	324(71.2%)	190(58.6%)		58(17.9%)	
Adjuvant chemotherapy					
No	167(36.7%)	102(61.1%)	0.692	30(18%)	0.999
Yes	287(63.1%)	169(58.9%)		51(17.8%)	
Anti HER2 targeted therapy					
No	343(75.4%)	205(59.8%)	0.912	58(16.9%)	0.395
Yes	112(24.6%)	66(58.9%)		23(20.5%)	

Abbreviations: ARD, Acute radiation dermatitis; BCS, Breast-conserving surgery; BMI, Body mass index; CFRT, Conventional fractionated radiotherapy; ER, Estrogen receptor; HER2, Human epidermal growth factor receptor 2; HFRT, Hypo-fractionated radiotherapy; PR, Progesterone receptor; TNBC, Triple-negative breast cancer

### Association between severe ARD and ferritin, hs-CRP, and lymphocyte subsets

The results of normal distribution show that all except CD4 + T cells are non-normal distribution (Supplementary table). Non-parametric test was used to explore the differences among different clinical characteristics of ferritin, hs-CRP and lymphocyte subsets. CD4 + T cells were tested by T test. As shown in the Table 2, age, BMI, hypertension, diabetes, smoking, adjuvant chemotherapy and anti-HER2 therapy affected the level of ferritin, hs-CRP and lymphocyte percentage. Whether the neoadjuvant chemotherapy, the mode of operation and other factors had no effect on these blood indexes.

**Table 2:** Significance test of distribution difference ferritin, hs-CRP and lymphocyte subsets levels by Clinical Characteristics

Variables	Ferritin *P*	hs-CRP *P*	CD3 + T *P*	CD4 + T P*	CD8 + T *P*	B *P*	NK *P*
Age	< 0.001	0.038	0.001	0.693	0.005	0.643	< 0.001
BMI	0.169	0.003	0.509	0.176	0.396	0.278	0.28
Hypertension	0.061	0.041	0.481	0.27	0.561	0.191	0.923
Diabetes	0.194	0.072	0.425	0.439	0.483	0.625	0.949
Smoking	0.008	0.134	0.327	0.501	0.468	0.495	0.675
Surgery	0.32	0.426	0.109	0.387	0.029	0.197	0.353
Neoadjuvant chemotherapy	0.195	0.118	0.542	0.242	0.01	0.002	0.036
Adjuvant chemotherapy	0.001	0.865	0.001	0.885	0.101	< 0.001	0.899
AntiHER2	0.324	0.609	0.004	0.756	< 0.001	0.236	0.035

*: CD4 + T cells were compared by independent t-test. The nonparametric test was used for other continuous variables

Abbreviations: hs-CRP, High-sensitivity C-reactive protein; CD3 + T, CD3 + T lymphocytes,%; CD4 + T, CD4 + T lymphocytes,%; CD8 + T, CD8 + T lymphocytes,%; B, B lymphocytes,%; NK, Natural killer lymphocytes,%

To explore the association of continuous variables, such as ferritin levels, hs-CRP levels, and the percentage of lymphocyte subsets and ARD, these variables were divided into four quartile groups. Table 3 lists the association between ARD severity with quartiles of variables. A significant relationship was discovered between ONS grade 3 + and grade 4 + ARD and increasing ferritin levels (P linear trend < 0.001). Hs-CRP levels were also linked to grade 3 + ARD (P linear trend = 0.015) and grade 4 + ARD (P linear trend < 0.001). Patients with the highest quartile of ferritin or hs-CRP had a significantly increased risk of developing grade 3 + and grade 4 + ARD. Patients with a CD3 + T lymphocytes percentage greater than or equal to 77.87% had an increased risk of developing severe ARD.

**Table 3:** Association Between variables and ARD severity

Variable in Quartiles		≧ 3 grade			≧ 4 grade		
		OR	95% CI	*P*	OR	95% CI	*P*
Ferritin,µg/L	< 40.60			< 0.001			< 0.001
	40.60 ~ 81.60	1.28	0.76–2.15	0.354	1.09	0.49–2.43	0.838
	81.60 ~ 154.40	1.53	0.91–2.58	0.112	1.36	0.63–2.95	0.434
	≧ 154.40	3.59	2.03–6.36	< 0.001	3.78	1.88–7.61	< 0.001
hs-CRP, mg/L	< 0.44			0.015			< 0.001
	0.44 ~ 1.03	1.46	0.84–2.52	0.178	0.51	0.21–1.26	0.146
	1.03 ~ 2.24	1.13	0.66–1.94	0.663	1.39	0.66–2.9	0.385
	≧ 2.24	2.44	1.37–4.34	0.002	3.02	1.53–5.98	0.002
CD3 + T lymphocytes,%	< 71.45			0.986			0.067
	71.45 ~ 77.87	0.99	0.58–1.68	0.957	2.02	0.92–4.43	0.081
	77.87 ~ 83.78	1	0.59–1.7	1	2.5	1.16–5.39	0.019
	≧ 83.78	0.92	0.54–1.56	0.744	2.67	1.24–5.72	0.012
CD4 + T lymphocytes,%	< 33.65			0.695			0.661
	33.65 ~ 39.86	1.24	0.72–2.11	0.443	1.4	0.69–2.85	0.354
	39.86 ~ 46.02	0.9	0.53–1.52	0.686	1.3	0.64–2.67	0.468
	≧ 46.02	1.08	0.63–1.83	0.786	1.55	0.77–3.12	0.22
CD8 + T lymphocytes,%	< 23.9			0.802			0.721
	23.9 ~ 28.70	1.01	0.6–1.72	0.964	1.11	0.57–2.15	0.765
	28.70 ~ 35.36	1.24	0.73–2.12	0.429	0.97	0.5–1.91	0.937
	≧ 35.36	0.97	0.57–1.64	0.91	0.74	0.36–1.5	0.402
CD4+/CD8 + ratio	< 1.00			0.842			0.498
	1.00 ~ 1.38	1.27	0.75–2.17	0.372	1.4	0.69–2.84	0.355
	1.38 ~ 1.85	1.08	0.64–1.83	0.763	1.26	0.62–2.58	0.522
	≧ 1.85	1.09	0.64–1.85	0.753	1.71	0.85–3.43	0.131

Abbreviations: Confidence interval; hs-CRP, High-sensitivity C-reactive protein; OR, Odds ratio; CI

### Univariate and multivariate analyses

In order to more easily predict the risk of severe ARD, we used the ROC curve to determine the best cut-off point of ferritin, hs-CRP, and lymphocyte subset levels (163.45 µg/L for ferritin, 1.305 mg/L for hs-CRP, 75.07% for CD3 + T lymphocytes, 41.705% for CD4 + T lymphocytes, 27.31% for CD8 + T lymphocytes, and 0.955 for the CD4+/CD8 + ratio) to divide the patients into lower and higher risk groups. A univariate analysis was conducted to screen risk factors for ONS 3+/4 + grade ARD. Table 4 indicates that patients with higher age, BMI, hypertension, diabetes, smoking history, ferritin levels, and hs-CRP levels were statistically associated with a higher risk of 3 + and 4 + grade ARD. Higher CD3 + T cell counts were also associated with a higher risk of 4 + grade ARD. A multivariate logistic regression was conducted including age, BMI, hypertension, diabetes, smoking history, adjuvant chemotherapy and anti-HER2 therapy, ferritin levels, hs-CRP levels, and CD3 + T cells. In multivariate logistic regression (Table 5), age (OR: 1.02, 95% CI: 1.004–1.05, P = 0.021) and higher ferritin levels (OR: 2.47, 95% CI: 1.37–4.46, P = 0.003) were independent prognosticators of 3 + grade ARD. After adjustment for other factors, BMI (OR: 1.11, 95% CI: 1.01–1.22, P = 0.033), diabetes (OR: 2.70, 95% CI: 1.11–6.60, P = 0.029), smoking history (OR: 3.04, 95% CI: 1.15–8.02, P = 0.025), higher ferritin levels (OR: 3.31, 95% CI: 1.78–6.17, P < 0.001), higher hs-CRP levels (OR: 1.96, 95% CI: 1.02–3.77, P = 0.044), and higher CT3 + T cells (OR: 1.99, 95% CI: 1.10–3.58, P = 0.022) remained statistically significant.

**Table 4:** Predictors of ARD severity in breast cancer patients: Univariate analysis

Variable	≧ 3 grade			≧ 4 grade		
	OR	95% CI	*P*	OR	95% CI	*P*
Age	1.79	1.05–3.06	0.034	1.74	0.97–3.13	0.064
BMI	1.14	1.07–1.22	< 0.001	1.24	1.15–1.34	< 0.001
Hypertension	3.09	1.66–5.75	< 0.001	3.95	2.25–6.94	< 0.001
Diabetes	4.77	2.10-10.86	< 0.001	5.1	2.74–9.49	< 0.001
Smoking	4.17	1.42–12.28	0.009	4.92	2.21–10.92	< 0.001
Stage I			0.198			0.472
Stage II	1.52	0.96–2.4	0.072	0.95	0.54–1.68	0.87
Stage III	1.27	0.78–2.07	0.335	0.69	0.36–1.32	0.262
ER	1.33	0.89–1.99	0.17	0.89	0.53–1.50	0.667
PR	1.22	0.83–1.79	0.305	0.79	0.48–1.28	0.333
HER2	1.06	0.69–1.62	0.807	1.24	0.73–2.10	0.433
TNBC	0.68	0.42–1.10	0.113	0.92	0.49–1.74	0.806
Ki67	0.92	0.58–1.45	0.705	0.95	0.53–1.70	0.869
Neoadjuvant chemotherapy	0.98	0.65–1.46	0.906	0.98	0.58–1.65	0.941
Surgery	0.88	0.58–1.33	0.53	1.02	0.6–1.74	0.931
Adjuvant chemotherapy	0.91	0.62–1.35	0.646	0.99	0.6–1.62	0.958
AntiHER2	0.97	0.63–1.49	0.875	1.27	0.74–2.18	0.384
Ferritin	2.6	1.57–4.30	< 0.001	4.15	2.48–6.94	< 0.001
hs-CRP	1.63	1.09–2.44	0.017	2.95	1.76–4.95	< 0.001
CD3 + T	1.03	0.70–1.50	0.9	2.15	1.25–3.67	0.005
CD4 + T	0.97	0.67–1.42	0.88	1.49	0.92–2.41	0.105
CD8 + T	1.18	0.81–1.71	0.402	1.1	0.67–1.78	0.71
CD4+/CD8 + ratio	1.11	0.70–1.74	0.664	1.75	0.91–3.38	0.096

Abbreviations: ARD, Acute radiation dermatitis; BCS, Breast-conserving surgery; BMI, Body mass index; CI, Confidence interval; CFRT, Conventional fractionated radiotherapy; ER, Estrogen receptor; HFRT, Hypo-fractionated radiotherapy; hs-CRP, High-sensitivity C-reactive protein; CD3 + T, CD3 + T lymphocytes; CD4 + T, CD4 + T lymphocytes; CD8 + T, CD8 + T lymphocytes; HER2, Human epidermal growth factor receptor 2; PR, Progesterone receptor; OR, Odds ratio; TNBC, Triple-negative breast cancer

**Table 5:** Predictors of ARD severity in breast cancer patients: Multivariate analysis

Variable	≧ 3 grade			≧ 4 grade		
	OR	95%CI	*P*	OR	95%CI	*P*
Age	1.02	1.004–1.05	0.021	1.002	0.97–1.03	0.892
BMI	1.06	0.98–1.15	0.162	1.11	1.01–1.22	0.033
Hypertension	1.67	0.77–3.63	0.199	1.85	0.87–3.92	0.108
Diabetes	2.12	0.8–5.61	0.128	2.7	1.11–6.6	0.029
Smoking	3.07	0.99–9.51	0.052	3.04	1.15–8.02	0.025
Adjuvant chemotherapy	0.85	0.55–1.34	0.488	1.02	0.54–1.92	0.945
Anti-HER2	1.03	0.63–1.69	0.906	1.63	0.84–3.16	0.15
Ferritin	2.47	1.37–4.46	0.003	3.31	1.78–6.17	< 0.001
hs-CRP	0.92	0.59–1.44	0.72	1.96	1.02–3.77	0.044
CD3 + T	1.16	0.75–1.8	0.516	1.99	1.1–3.58	0.022

Abbreviations: ARD, Acute radiation dermatitis; BMI, Body mass index; CI, Confidence interval; hs-CRP, High-sensitivity C-reactive protein; CD3 + T, CD3 + T lymphocytes; OR, Odds ratio

### Development and validation of the nomogram

**Fig. 1:**
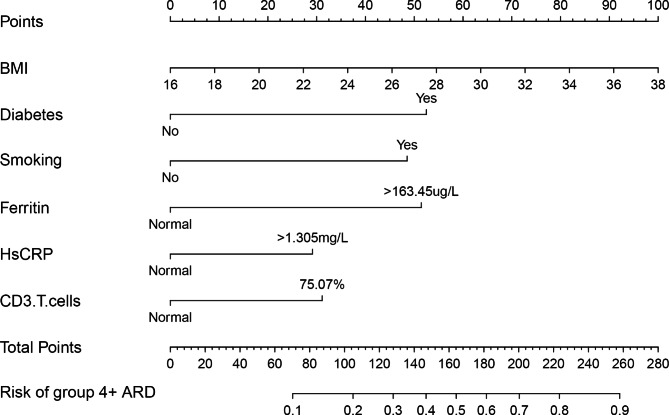
Nomogram predicting the risk of severe acute radiation dermatitis based on BMI, diabetes, smoking history, pre-radiotherapy ferritin, hs-CRP, and CD3 + T lymphocytes in breast cancer patients. Note: The first line is called points, which is the score reference of each variable. For each individual patient, six lines are drawn upward to determine the points received from the six variables in the nomogram. According to the individual parameters of each patient, the sum of these points is located on the ‘‘Total Points” axis, and a line is drawn downward to determine the likelihood of this patient to have 4 + ARD. For example, a breast cancer patient with BMI 27 who has diabetes and no history of smoking, whose serum ferritin is higher than 163.45/ug/L, hs-CRP and CD3+ T lymphocytes is normal, then the points of each parameter are 50, 52, 0, 53, 0, 0 respectively. The total score is 155, and the mapping to the lowest linear predictor is about 45%. As a result, the patient’s risk of developing ARD is 45%

Patients who have ARD grade 4 and higher, as determined by the ONS, have a much lower quality of life and a higher risk of secondary infections. Therefore, in this study, the multivariate logistic regression analysis results were used to construct a nomogram model to facilitate clinicians’ individualized prediction of the risk of grade 4 + ARD. Based on multiple logistic regression coefficients, the prediction model shown in Fig. 1 is visually presented in the form of a nomogram.

The scoring equation was established as follows: logit(p) = -5.544 +  0.106* BMI+1.228*diabetes + 1.136*smoking history + 1.204*ferritin + 0.682*hs-CRP + 0.729*CD3 + T cells. The risk of ONS Grade 4 + ARD was as follows: p = 1/(1 + exp[-logit(p)])

**Fig. 2:**
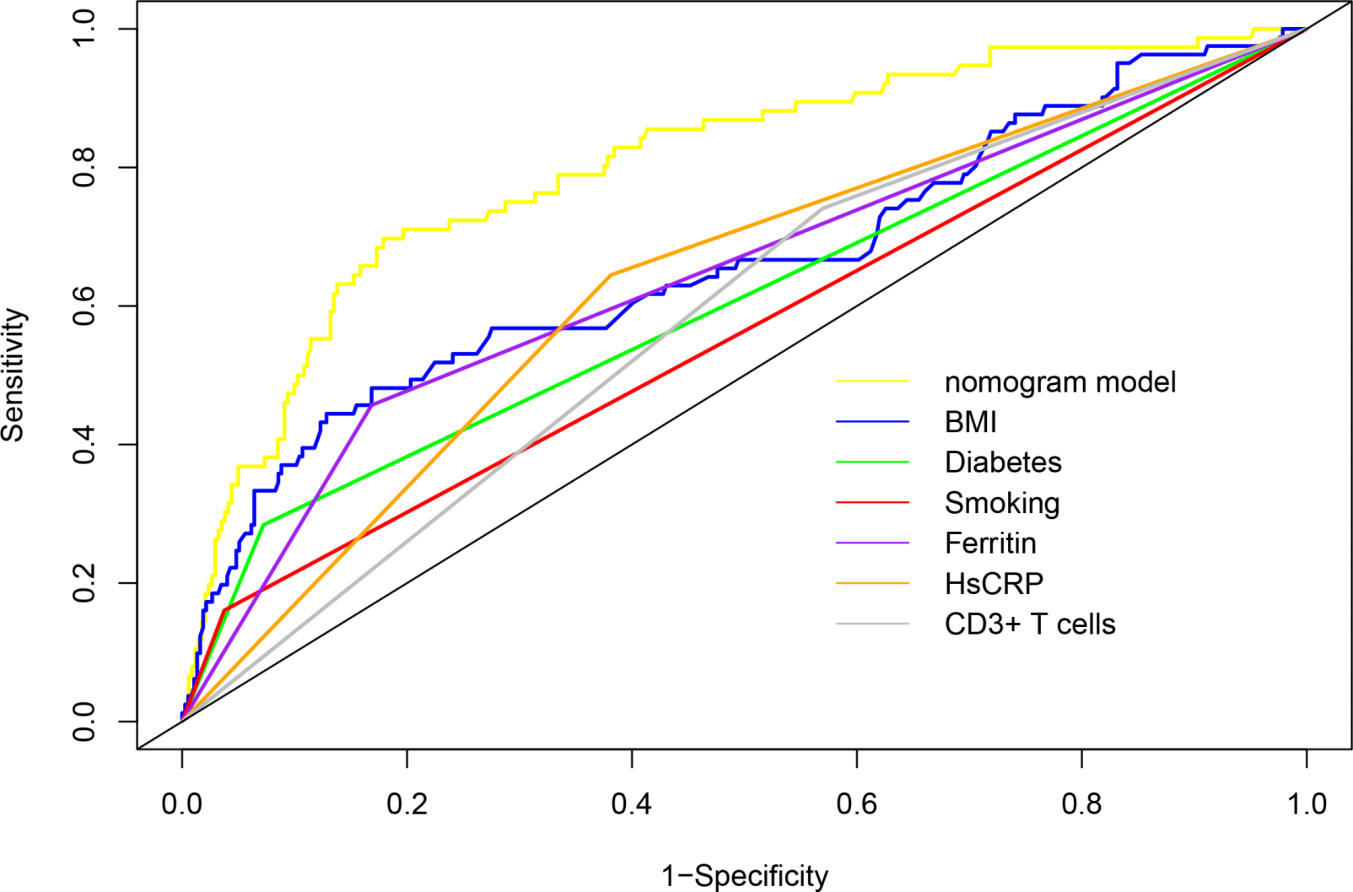
ROC curves of risk factors and the predictive model for grade 4 + ARD

**Fig. 3:**
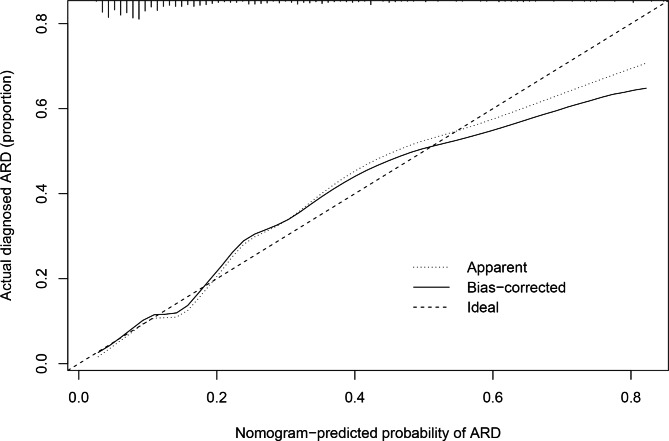
Calibration curves of the nomogram predicting the risk of grade 4 + ARD

**Fig. 4:**
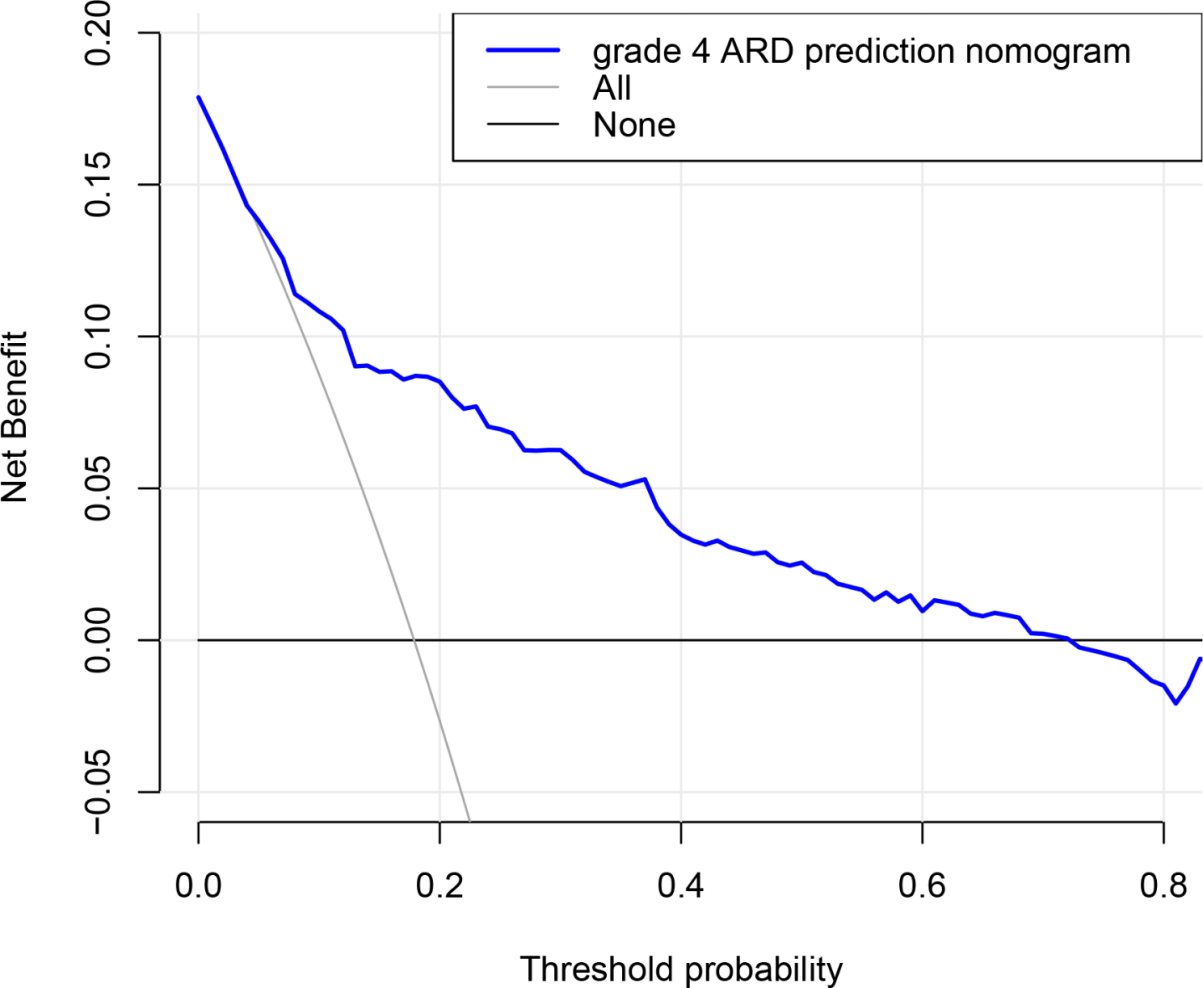
Decision curves of the nomogram predicting the risk of grade 4 + ARD

The ROC curves of BMI, diabetes, smoking history, higher ferritin levels, higher hs-CRP levels, CD3 + T cells, and the nomogram are shown in Fig. 2; Table 6. The predictive nomogram model had a ROC AUC of 0.80 (95% CI: 0.75–0.86), indicating that it had greater discriminative power than a single factor. The calibration curve (Fig. 3) shows that the nomogram predicted probabilities were consistent with the actual observed 4 + grade ARD. DCA for the nomogram is presented in Fig. 4, which shows that the nomogram can accurately predict 4 + grade ARD. This also indicated that the model had a potential clinical value.

**Table 6:** ROC analysis of the predictors and nomogram model in predicting ARD

Variables	AUC	95% CI
BMI	0.66	0.59–0.73
Diabetes	0.61	0.56–0.66
Smoking	0.56	0.52–0.60
Ferritin	0.64	0.59–0.70
Hs-CRP	0.63	0.57–0.69
CD3 + T	0.59	0.53–0.64
Normagram	0.80	0.75–0.86

Abbreviations: ARD, Acute radiation dermatitis; AUC, Area under the curve; BMI, Body mass index; CI, Confidence interval; hs-CRP, High-sensitivity C-reactive protein; CD3 + T, CD3 + T lymphocytes; OR, Odds ratio

## Discussion

[5][6]In this prospective cohort study, BMI, diabetes, smoking history, higher ferritin levels, higher hs-CRP levels, and higher CD3 + T lymphocytes before radiotherapy were found to be independent predictors of grade 4 + ARD in breast cancer patients undergoing RT. A nomogram model was also developed to predict the risk of grade 4 + ARD. The nomogram model’s internal validation proved its superiority to any single risk factor alone and its potential clinical value.

Large breasts and obesity have been found to increase the risk of ARD according to multiple observational studies [26]. BMI is closely related to breast volume [27]. Our results suggested that ARD risk increased with increasing BMI. Obesity is rapidly increasing globally and is now recognized as a cause of chronic subclinical inflammation. The link between BMI and the risk of ARD could be explained by the inhomogeneity of radiotherapy skin dose, skin fold friction, and easy sweating in obese patients. Additionally, obese people may be experiencing chronic inflammation, which makes them more prone to set off inflammation and lead to the development of ARD [28].

Our data confirmed prior research findings that smoking was a risk factor for developing ARD [26, 29, 30]. Although few studies have directly investigated the mechanism by which smoking causes ARD, it has been shown that smoking induces a pro-inflammatory response, oxidative stress, and skin microvascular dysfunction [31, 32]. Among patients with atopic dermatitis, smokers showed more pruritus, more exudation, and/or crusting than non-smokers [33]. In hemodialysis patients with uremic pruritus and elevated serum interleukin (IL)-31 levels, smoking is associated with moderate to severe pruritus [34, 35].

According to this study, the risk of ARD is significantly higher in patients who are older or have diabetes. There is disagreement in research reports about whether age affects post- radiotherapy toxic damage and the degree of damage [36–38]. Studies have reported no significant difference in median age between patients with severe radiation toxicity and those with mild symptoms, indicating that older patients tolerate radiotherapy well [36, 39]. However, a study comparing asymptomatic patients to those with severe ARD discovered that women over the age of 59 had a higher rate of severe ARD (OR = 6.6; p = 0.02) [40]. Diabetes mellitus is a metabolic disorder that has a negative impact on wound recovery and healing. Diabetes can also cause acute injury to progress to chronic injury, which provides a favorable environment for opportunistic pathogens and increases the risk of wound infection [41]. One study found a significant association between diabetes and the development of chronic ulcers in patients with ARD (Spearman: r = 0.86; P = 0.001) [6]. However, many of the patients with diabetes, hypertension, and hyperlipidemia were also taking blood sugar-lowering drugs or statins. Metformin and gliclazide have been reported to exert radioprotective effects on human cells [42, 43], and there is evidence that statins may accelerate DNA repair and reduce the expression of proinflammatory cytokines [44, 45]. Further research is needed to determine whether these factors are related to ARD and whether they have an effect on the repair mechanism of skin radiation damage.

Radiation damage is a complex multifactorial process involving multiple biological pathways. Previous studies have suggested that radiation-induced changes in pro-inflammatory cytokines and growth factors may contribute to normal tissue toxicity [46, 47]. At present, many studies have shown that the inflammatory index CRP is related to ARD severity [16, 48]. Ferritin is an iron storage protein, and it is also a well-known inflammatory marker that can be significantly increased in acute and chronic inflammation [17]. Studies have reported that ferritin can act as a pro-inflammatory signaling molecule by activating the phosphorylation of PI3 kinase, protein kinase C, MAP kinase, and NFκB pathways, which further promotes the expression of pro-inflammatory mediators (e.g., IL-1β, inducible nitric oxide synthase, intercellular adhesion molecule 1, etc.) and aggravates the inflammatory response [18]. Our results suggested that the elevations of ferritin and hs-CRP before radiotherapy were associated with the occurrence of grade 4 + ARD, which may indirectly reflect the inflammatory stress state in patients before radiotherapy. Elevated plasma hs-CRP and ferritin levels are associated with diseases such as cancer prognosis, insulin resistance, and type 2 diabetes, which may also affect overall survival [49, 50]. Therefore, patients with elevated hs-CRP and ferritin levels should be actively monitored in clinical work for a variety of diseases that may affect overall survival.

Skin reactions are closely related to the radiation dose received by the skin [29]. Patients after total mastectomy receive higher doses to the skin when irradiating the chest wall. Therefore, patients after total mastectomy are considered to be at a higher risk of developing severe ARD than patients after BCS. However, our study found no statistically significant difference between the occurrence of grade 4 + ARD and breast cancer surgery. In this study, strict quality control of radiotherapy plans was included constraints for skin volume and limiting the proportion of hot spots. One study also found that the mean PTV of BCS patients was larger than that of total mastectomy patients (790 mL versus 580 mL, respectively), and they suspected that a larger PTV might increase the incidence of severe ARD [51]. In addition, these patients received boost irradiation on the tumor bed after BCS, which was considered one of the risk factors for severe ARD.

The advantages of the current study are mainly as follows: (1) The prospective cohort design has a certain degree of objectivity and reduces retrospective bias. (2) A nomogram model was developed based on the multivariate logistics regression model to predict the risk of severe ARD in individual patients, allowing for individualized risk prediction. However, this study has some limitations. ROC curve analysis, calibration curve model, and DCA curves were used to evaluate the constructed nomogram model. However, in order to make the predictive model more clinically relevant, its performance needs to be further validated in a validation cohort. There could be potential risk factors that were not included in the study, such as genetic markers and skin radiation dose. In addition, the small number of patients exposed to certain study factors, such as smoking, age ≥ 60 years old, diabetes, etc., can cause some bias in the assessment of the association of these factors with ARD. Finally, this study was conducted in a single-center study in China. Similar studies in additional centers in China and other countries are needed to determine the clinical relevance of our nomogram in a wider setting.

## Conclusion

According to the ONS assessment criteria, 17.8% of breast cancer patients developed grade 4 + ARD (wet desquamation) after radiotherapy. BMI, diabetes, smoking history, higher ferritin levels, higher hs-CRP levels, and higher CD3 + T cells before RT were independent risk factors for grade 4 + ARD. The nomogram model based on the aforementioned risk factors has high prediction accuracy and certain clinical value. Based on the nomogram, clinicians can assess the risk of severe ARD before RT, adjust the mode of RT in advance (such as choosing HFRT), and take preventive and treatment measures (such as using topical corticosteroids, antiperspirant, washing with water and soap), which will reduce the risk of severe ARD.

## Electronic supplementary material

Below is the link to the electronic supplementary material.

Supplementary Material 1

## Data Availability

All data generated or analyzed during this study will be made available by the corresponding authors, upon reasonable request.

## Abbreviations

ARD: Acute radiation dermatitis
AUC: Area under the curve
BCS: Breast-conserving surgery
BMI: Body mass index
CFRT: Conventional fractionated radiotherapy
CI: Confidence interval
CTCAE: Common Terminology Criteria for Adverse Events
DCA: Decision curve analysis
ER: Estrogen receptor
HER2: Human epidermal growth factor receptor 2
HFRT: Hypo-fractionated radiotherapy
hs-CRP: High-sensitivity C-reactive protein
IMRT: Intensity modulated radiation therapy
NK: Natural killer
ONS: Oncology Nursing Society
OR: Odds ratio
PR: Progesterone receptor
RILA: Radiation-induced lymphocyte apoptosis
ROC: Receiver operating characteristic
RT: Radiotherapy
TNBC: Triple-negative breast cancer
