# Supplementary material for: Predicting acute radiation dermatitis in breast cancer: a prospective cohort study
Source: BMC Cancer. 2023 Jun 12;23:537. doi: 10.1186/s12885-023-10821-6 (PMC10262502; doi:10.1186/s12885-023-10821-6)
Supplement: Supplementary file 1 — Supplementary Material 1 [file 12885_2023_10821_MOESM1_ESM.docx]

Supplementary data: test results of normal distribution for ferritin, CRP and lymphocyte subsets.

| Histogram | P-P diagram | Q-Q diagram |
| --- | --- | --- |
| 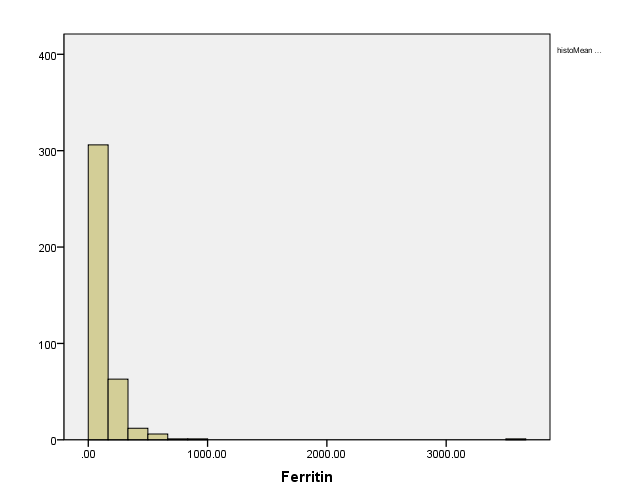 | 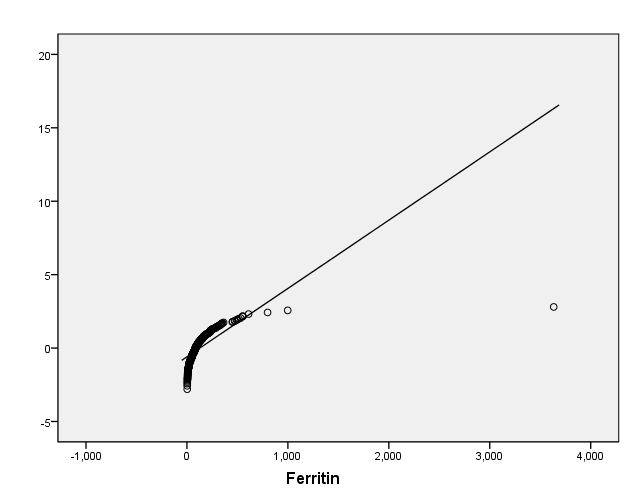 | 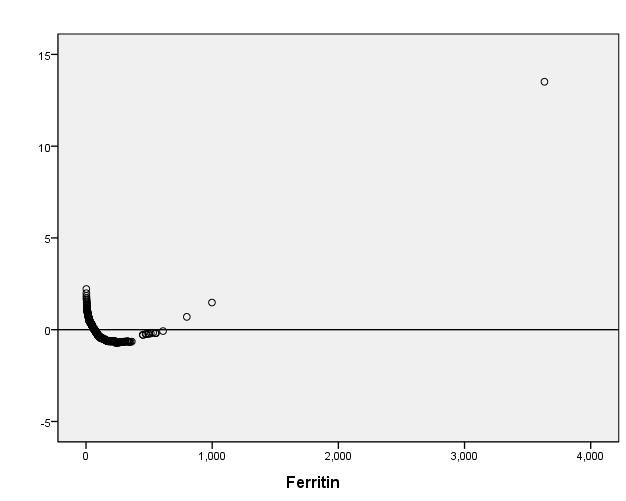 |
| 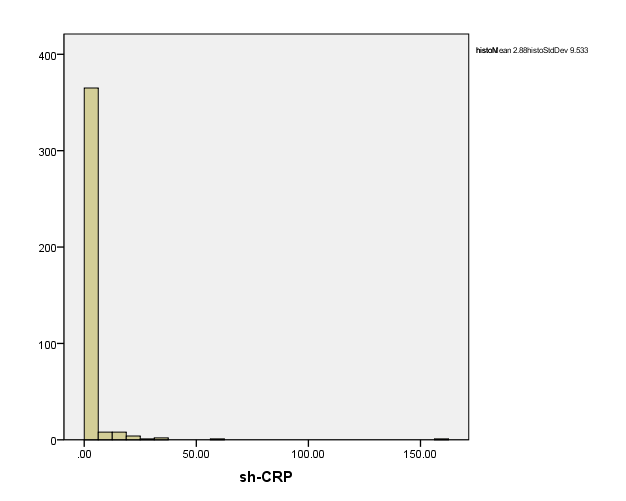 | 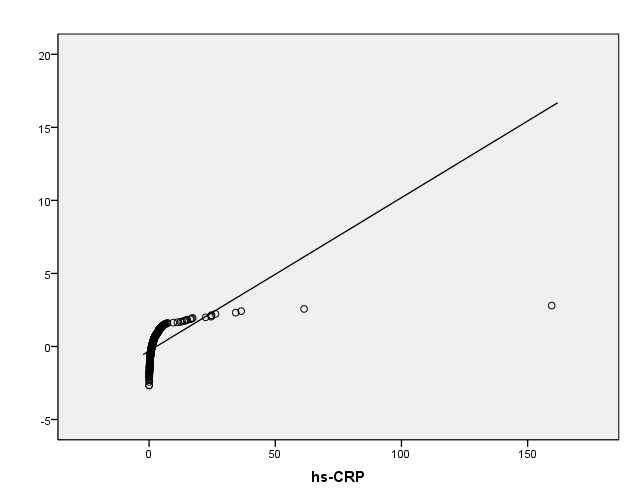 | 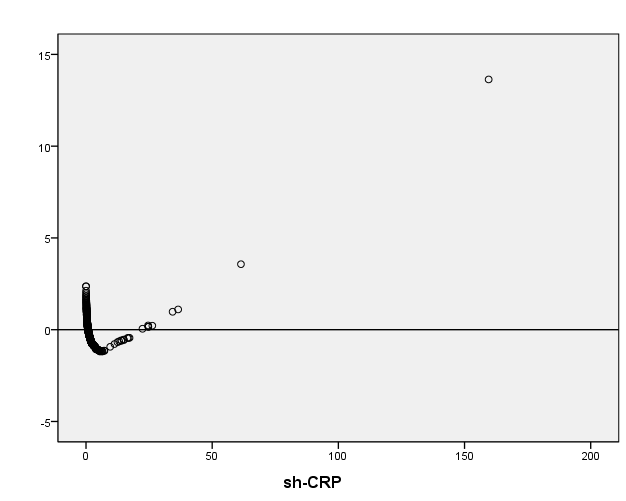 |
| 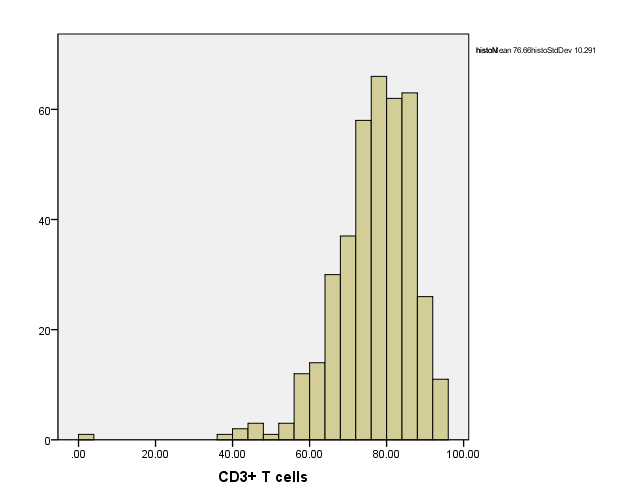 | 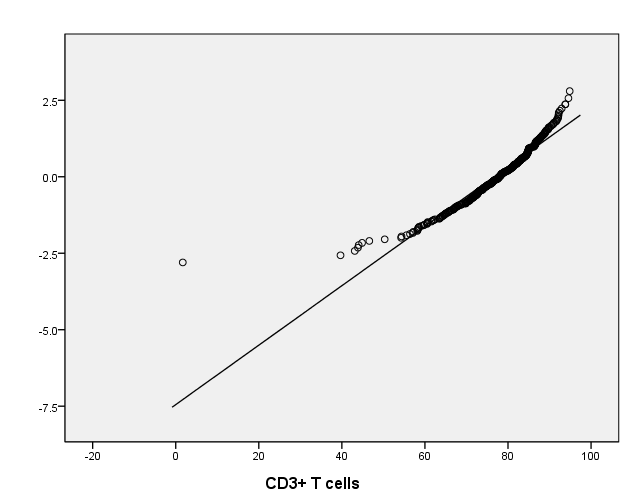 | 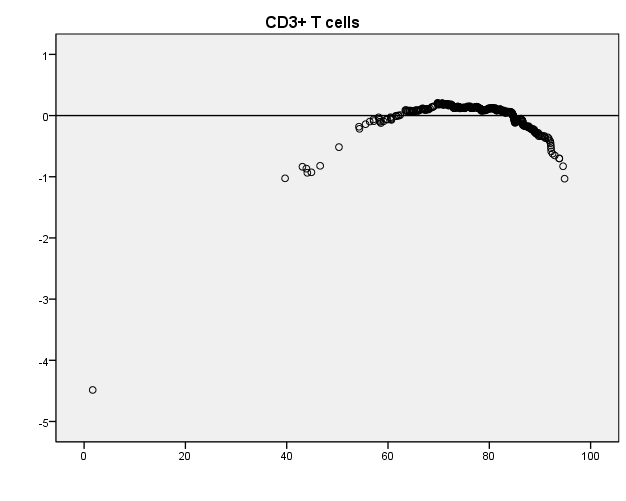 |
| 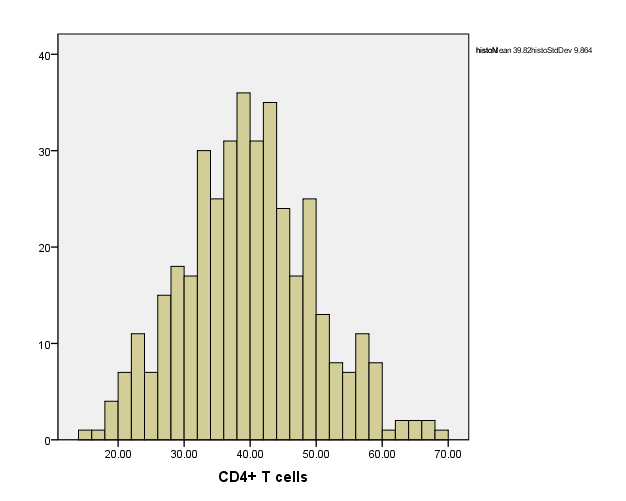 | 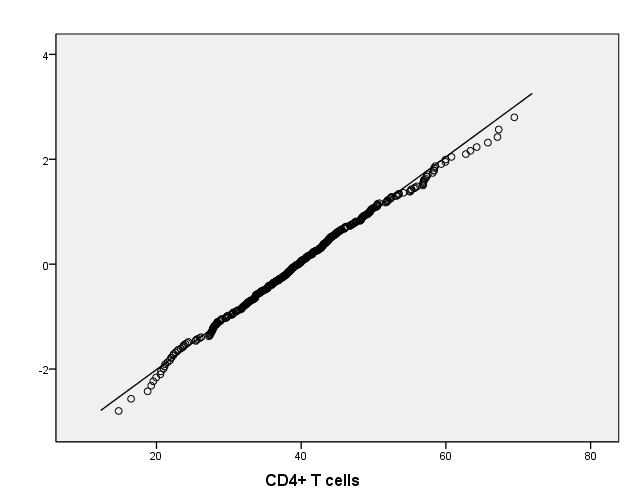 | 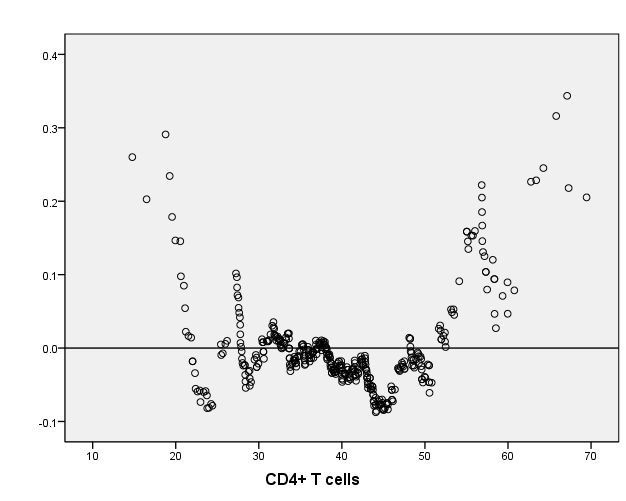 |
| 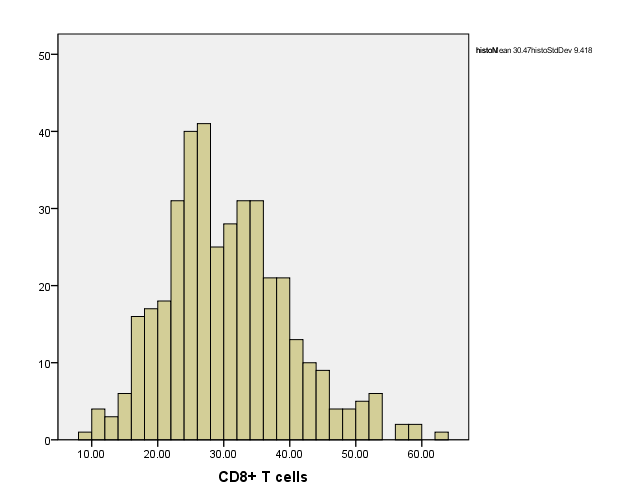 | 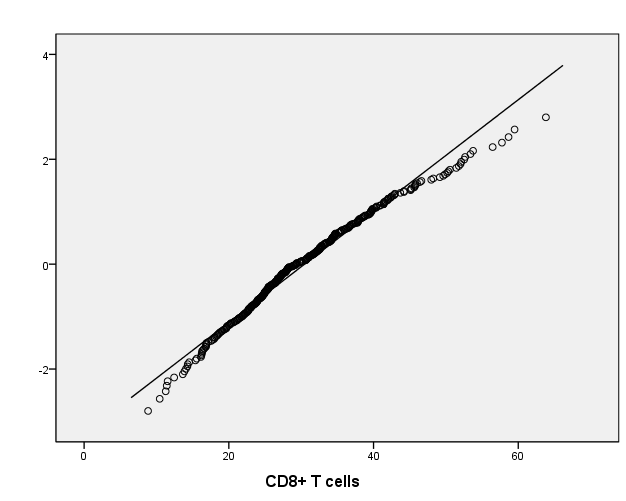 | 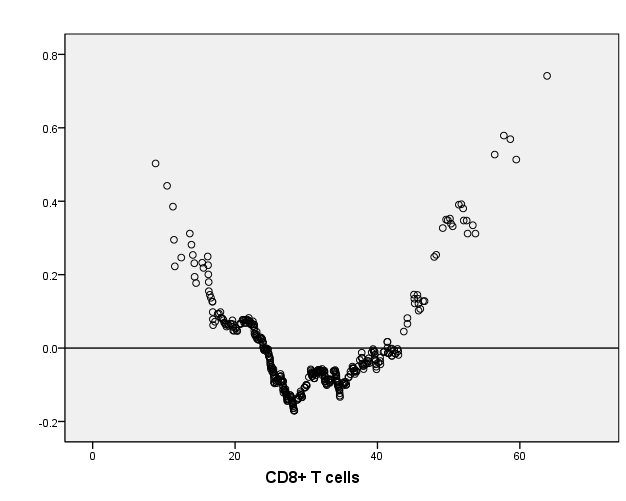 |
| 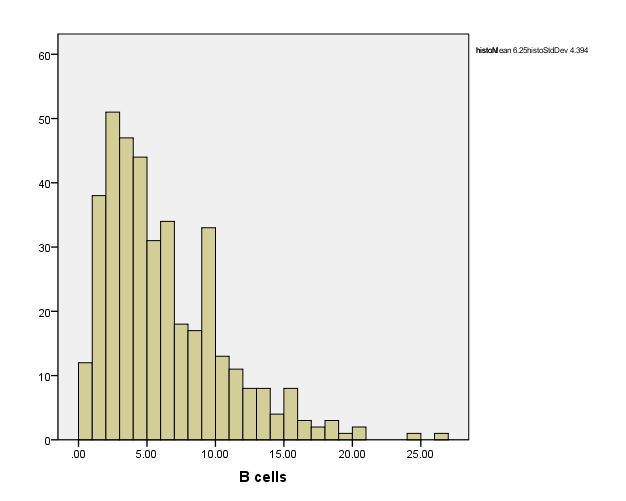 | 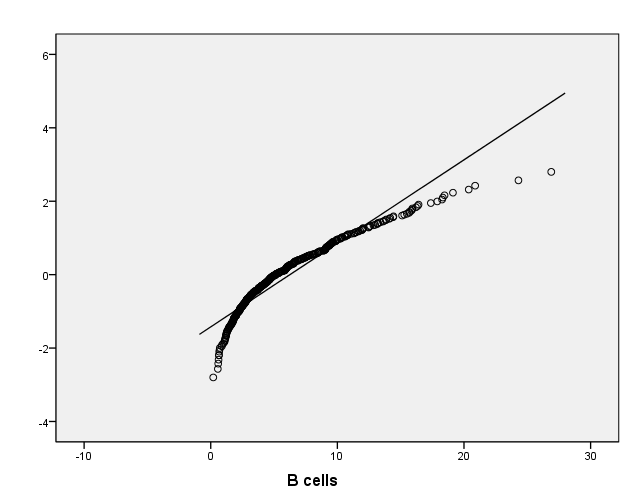 | 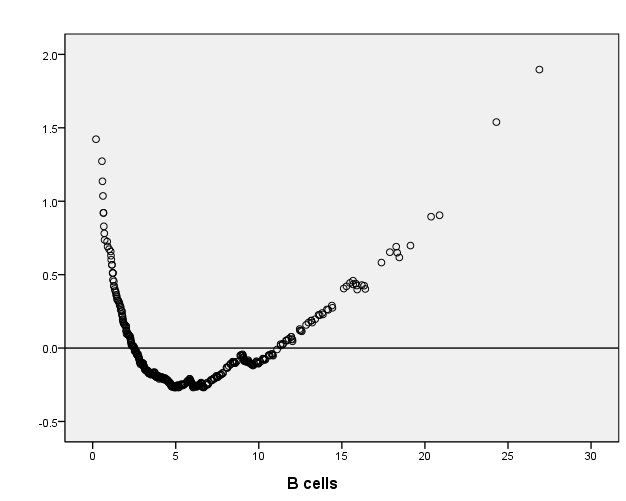 |
| 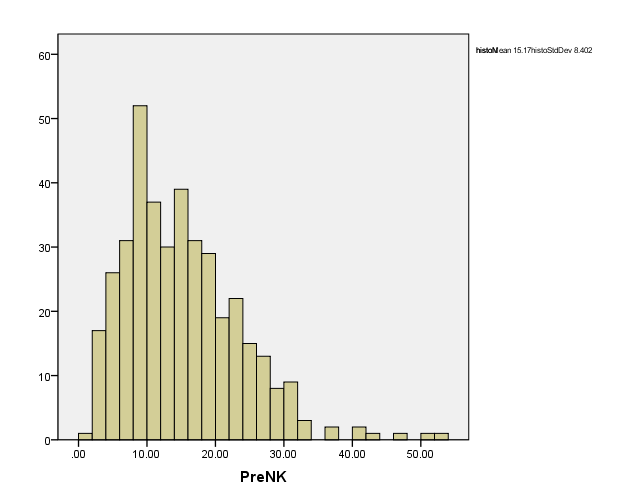 | 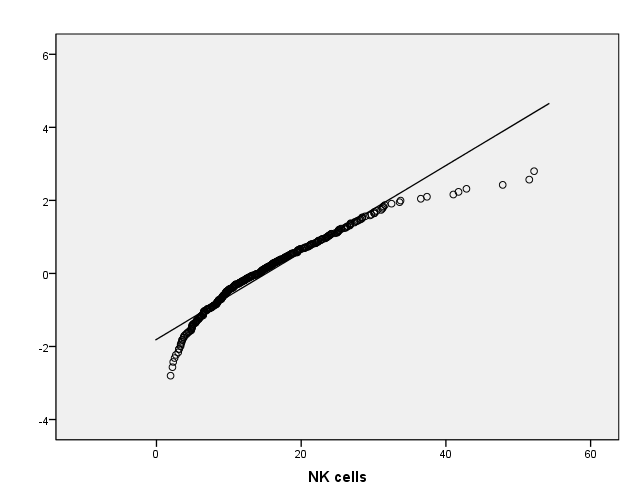 | 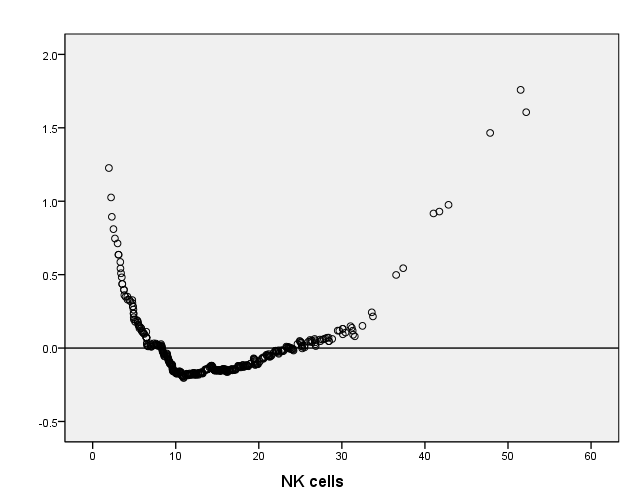 |
